# Metabolomic and exposomic biomarkers of risk of future neurodevelopmental delay in human milk

**DOI:** 10.1038/s41390-022-02283-6

**Published:** 2022-09-15

**Authors:** Kefeng Li, Kerri Bertrand, Jane C. Naviaux, Jonathan M. Monk, Alan Wells, Lin Wang, Sai Sachin Lingampelly, Robert K. Naviaux, Christina Chambers

**Affiliations:** 1 The Mitochondrial and Metabolic Disease Center, University of California, San Diego School of Medicine, San Diego, CA USA; 2 Department of Medicine, University of California, San Diego School of Medicine, San Diego, CA USA; 3 Department of Pediatrics, University of California, San Diego School of Medicine, San Diego, CA USA; 4 Department of Neurosciences, University of California, San Diego School of Medicine, San Diego, CA USA; 5 Department of Bioengineering, University of California, San Diego School of Medicine, San Diego, CA USA; 6 Department of Pathology, University of California, San Diego School of Medicine, San Diego, CA USA; 7 Herbert Wertheim School of Public Health and Human Longevity Science, University of California, San Diego School of Medicine, San Diego, CA USA

## Abstract

**Background:**

The chemical composition of human milk has long-lasting effects on brain development. We examined the prognostic value of the human milk metabolome and exposome in children with the risk of neurodevelopmental delay (NDD).

**Methods:**

This retrospective cohort study included 82 mother–infant pairs (40 male and 42 female infants). A total of 59 milk samples were from mothers with typically developing children and 23 samples were from mothers of children at risk. Milk samples were collected before 9 months of age (4.6 ± 2.5 months, mean ± SD). Neurocognitive development was assessed by maternal report at 14.2 ± 3.1 months using the Ages and Stages Questionnaires-2.

**Results:**

Metabolome and exposome profiling identified 453 metabolites and 61 environmental chemicals in milk. Machine learning tools identified changes in deoxysphingolipids, phospholipids, glycosphingolipids, plasmalogens, and acylcarnitines in the milk of mothers with children at risk for future delay. A predictive classifier had a diagnostic accuracy of 0.81 (95% CI: 0.66–0.96) for females and 0.79 (95% CI: 0.62–0.94) for males.

**Conclusions:**

Once validated in larger studies, the chemical analysis of human milk might be added as an option in well-baby checks to help identify children at risk of NDD before the first symptoms appear.

**Impact:**

- Maternal milk for infants sampled before 9 months of age contained sex-specific differences in deoxysphingolipids, sphingomyelins, plasmalogens, phospholipids, and acylcarnitines that predicted the risk of neurodevelopmental delay at 14.2 months of age.
- Once validated, this early biosignature in human milk might be incorporated into well-baby checks and help to identify infants at risk so early interventions might be instituted before the first symptoms appear.

## Introduction

Neurodevelopmental delay (NDD) refers to the delays in skill development of infants and young children, including physical, linguistic, behavior, or learning areas.^1^ According to a survey conducted in the United States for the years from 2009 to 2017, about 17% of children between the ages of 3–17 were affected by neurodevelopmental disorders, and recent trends have shown a significant increase in the prevalence of autism spectrum disorder since 2009.^2^ Treatment of neurodevelopmental disorders can be difficult since some neurodevelopmental disorders cannot be confidently diagnosed until children are 2–3 years of age. Some symptoms, once developed, may continue throughout life, causing enormous social and economic burdens. Children diagnosed with neurodevelopmental disorders have better treatment outcomes if detected and treated at younger ages.^3^ Therefore, new methods for early prediction of an infant’s later neurodevelopmental risk are urgently needed.

Early infancy is a critical period for neurodevelopment. As the ideal nutrition recognized by the World Health Organization for infants, human milk (HM) can have lasting effects on the structure and function of the developing brain. Compelling evidence supports the overall beneficial effects of HM intake on neurodevelopment.^4–6^ However, limited information is available about the impact of milk composition on breastfed infants’ neurocognitive outcomes.

Compositionally, human milk contains various amounts of protein, fat, carbohydrates, amino acids, and other metabolites, prebiotic factors like HM oligosaccharides, hormones, peptide growth factors, cytokines, microRNAs, minerals, and microbiota.^7,8^ A recent study reported that protein content in HM is not correlated with preterm infant neurodevelopment at 2 years of age.^9^ Metabolites in HM are the nutritional building blocks that support physical growth, immune system development, and brain maturation for infants.^10,11^ The associations between metabolites in HM and the neurodevelopment and cognitive function in young offspring have only been sparsely examined and primarily focused on the total fat content and long-chain polyunsaturated fatty acid in HM.^12,13^ For example, a recent systematic review has shown the association between fat content in HM for premature infants and neurodevelopmental outcomes.^14^ In addition, higher linolenic acid and docosahexaenoic acid was found in milk collected at the third month of life were reported to be correlated with better psychomotor development of exclusively breastfed infants at the sixth month of life.^15^ However, total fat concentration does not reflect different lipid classes and other endogenous metabolites in HM, which also appears to be important for infant growth.^8^ Milk composition varies somewhat over the course of a day.^16^ For example, the fat content varies from about 4.1–5.2% over a day,^17^ and the time since the last feeding can influence the composition of milk available in the next feeding.^18^ No studies have yet been published on the comprehensive analysis of the metabolomic differences in human milk between typically developing (TD) children and children with NDD.

A mother is exposed to various environmental chemicals through food, personal care products, household products, medical and recreational drugs, pollutants, or through her occupational environment. Some of this maternal body burden can be transferred to HM during lactation.^19^ Infant early life exposure to environmental chemicals through milk is uniquely influential to their long-term outcomes due to heightened neuroplasticity during early childhood sensitive periods.^20^ Experimentally, several common classes of environmental chemicals in HM have been assessed for their associations with neurodevelopmental outcomes of the offspring.^21^ For example, in a mother–infant cohort from Vietnam, the neurodevelopmental scores for children at 4-month-old and 2-year-old were decreased with the increase in dioxin (2,3,7,8-tetrachlorodibenzo-p-dioxin) in HM collected at 1 month.^22,23^ The level of diethylhexyl phthalate in HM collected at 30 days after delivery was associated with impaired mental development in children aged 1–2 years.^24^ Scores for cognitive function were also lower in children who were exposed to higher amounts of the flame retardant brominated diphenyl ether-209 (BDE209) through HM.^25^ However, most of the existing epidemiological studies for neurodevelopmental effects of HM have only focused on a small set of environmental chemicals and hence may exclude important chemicals from consideration. Since the general population can be simultaneously exposed to a wide range of environmental chemicals, a comprehensive characterization of the human milk exposome will allow an in-depth evaluation of the potential long-term neurodevelopmental effects of the HM exposome.

In this study, we conducted integrated metabolomic and exposomic profiling of HM. This broad-based approach combined with multiple machine learning (ML) algorithms aimed to (1) determine the potential metabolomic and exposomic differences in HM for infants with and without NDD risk, and (2) develop and evaluate multi-analyte models using HM metabolome and exposome data to predict infants with the future risk of NDD.

## Materials and methods

### Study participants and sample collection

The study population is based on the subjects from the University of California, San Diego (UC San Diego) Human Milk Biorepository, which contains over 5900 HM samples from over 2200 women who reside in the U.S. and Canada along with subsequent neurodevelopmental screening assessments for their infants/children. We first excluded maternal participants who reported diabetes, depression, other medical conditions, or pregnancy complications. HM samples were collected using a standardized protocol previously described.^26^ Inclusion criteria for this study were milk samples collected from mothers of infants less than 9 months of age and the valid completion of an Ages and Stages Questionnaire version 2 (ASQ2) during follow-up of the infant between 12 and 25 months of age. Exclusion criteria included two or more conflicting ASQ2 communication scores above and below the age-specific developmental threshold for a given child. HM samples were either collected at the participant’s home and shipped on ice within 24 h of sample collection or collected on-site at UC San Diego and processed in the laboratory within 3 h of sample expression. Selected HM samples were aliquoted and stored at −80 °C before transfer to the Naviaux lab for analysis. All the study protocols were approved by the Institutional Review Board (IRB) at UC San Diego, and written consent was obtained upon the collection of the HM samples under Dr Chamber’s UC San Diego IRB-approved protocol #130658. Metabolomics and exposomics were conducted on deidentified samples under Dr Naviaux’s UC San Diego IRB-approved protocol #140072. Metabolomic and exposomic analysis of the samples was conducted from July 21, 2020, to September 22, 2020. Detailed participant and sample characteristics are summarized in Supplementary Table S1.

### Neurodevelopmental screening

Screening for neurocognitive abilities of the breastfed children was assessed by maternal reports using the ASQ2. ASQ2 is a parent/caregiver screening instrument that pinpoints developmental progress in children between the ages of one month and 5.5 years. The ASQ screening questionnaire has been found to have 84% overall agreement with validated tools such as the Bayley Scales of Infant Development.^27,28^ The overall sensitivity of the screening instrument for correctly identifying children with delays and the specificity of the instrument for identifying TD children are 72 and 88%, respectively.^27^ The questionnaire encompasses the domains of communication, gross motor, fine motor, problem solving, and personal social. Scores by domain are computed and compared to established cut-offs by child age and sex to identify areas of concern. For purposes of this study, children were classified as “screen positive for developmental delay” if they had an ASQ2 score that was critical or borderline in any single domain of the assessment. No genetic or biochemical data were available from the infants who tested at risk by ASQ2 score. Children were classified as “typically developing” (TD) if their scores in all domains were above the respective cutoff scores.

### Metabolomic and exposomic analysis

HM samples were thawed on ice and mixed with a set of internal standards. The endogenous metabolites and xenobiotic compounds were extracted with four volumes of 100% ethanol prechilled at −20 °C. Targeted, broad-spectrum, metabolomic analysis of endogenous metabolites was performed using a UFLC XR HPLC system (LC-20AD, Shimadzu) coupled with a Qtrap 5500 triple quadrupole mass spectrometer (SCIEX) (LC-MS/MS) as described earlier^29,30^ with minor modifications to expand the list of targeted chemicals. To acquire comprehensive metabolomic profiles, we performed LC-MS/MS analysis in both hydrophilic interaction liquid chromatography (HILIC-MS/MS) mode and reverse phase (RP-MS/MS) mode. A total of 659 endogenous metabolites covering all major human metabolic pathways and diverse chemical classes were targeted. We used a Shodex polymer-based aminopropyl column (HILIC mode) for most endogenous metabolites and a Raptor biphenyl column (RP mode) for some of the lipophilic metabolites that cannot be measured well by the HILIC method, such as gangliosides, deoxyceramides, phosphatidylcholines, some sphingomyelins, and steroid hormones.

Exposome profiling of xenobiotic chemicals in HM was conducted using both RP-MS/MS and gas chromatography (GC)-MS/MS methods based on the chemical properties of the targets. A total of 937 anthropogenic compounds were targeted, including 145 analytes on the RP-MS/MS platform and 792 chemicals on the GC-MS/MS platform. These xenobiotics were selected to broadly monitor human exposure to commonly used pesticides, food additives, personal care products, pharmaceuticals, persistent organic pollutants, phthalates, and flame retardants in the US. MRM transitions and the retention time (RT) were optimized using the purified standards, and two MRMs transitions were used for each analyte. The full targeted list and the associated MS/MS parameters and RTs are listed in Supplementary Tables S2 and S3. See Supplementary Methods for additional details.

### Comparison with the existing human milk metabolome data

To compare the HM endogenous metabolites detected in our study with known HM metabolome data, we downloaded the curated HM metabolites from the milk composition database and human metabolome database (HMDB). We also conducted an extensive literature review of known HM metabolites using the text-mining tool PolySearch.^2,31^ All compounds were mapped to standard chemical identifiers such as PubChem, CAS, and HMDB using the metabolite ID conversion tool in MetaboAnalyst 5.0, and the data harmonization results were manually checked. A web application DeepVenn was then used for the comparison and visualization of two lists.

### Statistical analyses

All general analyses were performed using GraphPad Prism 9.3.1 unless specified. Data were presented as the number n and percent (%) for categorical variables and median (IQR) or mean (±standard deviation) for continuous variables. Differences between TD and risk groups were assessed by Student’s *t*-test (parametric), Mann–Whitney *U* test (non-parametric) for continuous variables, and Fisher’s exact test for categorical variables. A two-tailed *P* value < 0.05 was considered statistically significant. Sample size calculations for future validation studies were performed using Pearson’s correlation-based effect size of 0.25, two-sided *α* of 0.05, and *β* value of 0.8 (https://sample-size.net/correlation-sample-size/).

### Metabolome-exposome inter-omics correlation networks

Spearman’s rank correlation was used to build the correlations between HM metabolome and exposome data using the processed *z*-scores. Spearman correlation *r* values, false discovery rate–adjusted *P* values, and *q* values were generated with all chemical pairs. Only correlations between each pair variable with absolute *r* > 0.5, and *q* < 0.05 were used to construct the final correlation networks by MetScape 3.0, a plug-in for Cytoscape 3.8.2.^32^

### Multi-analyte predictive model construction and evaluation

The predictive performance was evaluated using the area under the receiver operator characteristic (AUROC) curve and nomograms were also created using R 4.0.5 with corresponding R packages. Confidence intervals (CI) for the ROC curves were calculated by bootstrap resampling. The models were validated by permutation analysis (1000 times). Sensitivity, specificity, and accuracy were calculated by 2 × 2 contingency table analyses. Matthews correlation coefficient (MCC) was also calculated, which provided complementary information to common binary classification metrics, especially for class-imbalanced datasets.^33^ See details in the Supplementary Methods.

## Results

### Participant and sample selection

**Fig. 1:**
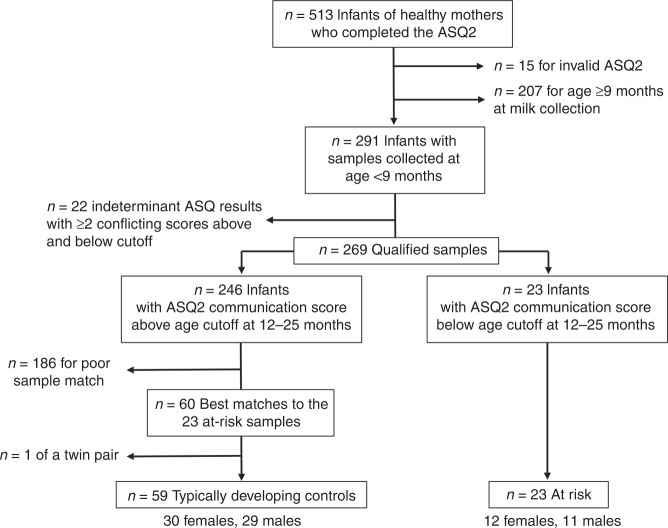
Participant and sample selection. From the records of 513 milk samples stored as part of the UCSD Human Milk Biorepository, 269 infant-sample pairs met inclusion and exclusion criteria. From the milk samples collected before the infants were 9 months old, metabolomic and exposomic analysis was performed on 59 control and 23 at risk samples. Developmental screening was performed by ASQ screening at 1–2 years of age. ASQ2 Ages and Stages Questionnaire version 2.

Figure 1 illustrates the participant and sample selection. After a bioinformatic review of all frozen and archived samples contained in the human milk biobank at the University of California, San Diego, we identified 269 qualified milk samples that met the inclusion and exclusion criteria for this study. From these samples, we identified 23 samples from infants who were found to be at risk for NDD by ASQ2 testing between 12 and 25 months of age. We next matched TD control samples to the at risk (AR) samples by frozen storage time, infant sex, and age at the time of milk sample collection and selected the 60 best-matched samples as controls for metabolomic and exposomic analysis. One male control infant was excluded to avoid genetic ascertainment bias because his twin sister qualified as a match. Participant and sample characteristics are summarized in Supplementary Table S1.

### Study population

Our study cohort included 82 mother–infant pairs, out of which 40 infants were male, and 42 were female (Table 1). Mothers were primarily non-Hispanic (91.5%), Caucasian (85.4%) with an annual household income ≥US$60,000 (80.5%), and already had a child (59.8%) (Table 1). At the time of milk sample collection, the mean maternal age was 33.7 ± 4.4 years old, and the mean age of the infants was 4.6 ± 2.5 months. The participants were periodically followed-up, and at the time of ASQ2 assessment (mean child age of 14.2 months), 23 children screened positive for NDD (the Risk group), while 59 were classified as TD. The time interval between milk collection and ASQ2 test was 9.5 (7.1–11.0) months (median and IQR). There was a significant difference in ASQ2 normalized scores between the risk and TD groups for the risk group, *P* < 0.0001 (Table 1). No significant differences were observed for other clinical variables between the risk and TD groups such as mother’s age, body mass index, gestational age at delivery, and infant birth weight (Table 1).

**Table 1:** Participant and sample characteristics.

	At risk, mean ± SEM (range)	Controls, mean ± SEM (range)	*P* value
Subjects enrolled	23	59	–
Sex	11 males	29 males	–
	12 females	30 females	
Gestational age at delivery (weeks)	37.6 ± 0.6 (28–40)	38.6 ± 0.3 (28–41)	ns
Maternal age (years)	34.6 ± 1.1 (23–46)	33 ± 0.4 (23–39)	ns
Maternal BMI (kg/m^2^)	26.2 ± 1.2 (19.1–40.7)	25.7 ± 0.6 (18.8–42.0)	ns
Parity (percent >1 child)	35% (8 of 23)	49% (29 of 59)	ns^a^
Percent preterm infants (<36 weeks)	13% (3 of 23)	3.4% (2 of 59)	ns^a^
Percent C-section delivery	26% (6 of 23)	29% (17 of 59)	ns^a^
Percent singleton pregnancies	96% (22 of 23)	97% (57 of 59)	ns^a^
Birth weight (g)	2950 ± 174 (1040–4139)	3311 ± 72 (1110–4507)	ns^b^
1-min APGAR score	7.8 ± 0.4 (4–9)	7.8 ± 0.2 (3–9)	ns
5-min APGAR score	8.7 ± 0.2 (7–9)	8.9 ± 0.1 (7–10)	ns
Infant age at time of milk collection (months)	4.1 ± 0.6 (0.4–8.3)	4.8 ± 0.3 (0.7–8.9)	ns
Time of day of milk collection (24-h clock)	1248 ± 46 (0730–2300)	1306 ± 29 (0645–2400)	ns
Milk sample storage time at −80 °C (years)	2.8 ± 0.1 (2.2–3.8)	3.0 ± 0.1 (1.9–5.1)	ns
Age at ASQ2 testing (months)	14.2 ± 0.7 (7.4–25.4)	14.2 ± 0.4 (12–25.4)	ns
Interval between milk collection and ASQ2 testing (months)	10.2 ± 0.8 (4.0–24.4)	9.5 ± 0.5 (4.5–21.9)	ns
Percent with ≥2 ASQ2 tests administered before 25 months	4.3% (1 of 23)	6.8% (4 of 59)	ns^a^
Age-normalized ASQ2 communication (*Z*-score)	−1.9 ± 0.2 (−3.2 to −0.92)	0.18 ± 0.1 (−1.8 to +1.0)	0.0001
Annual household income			
<$10,000	0	1	ns
$10,001–$59,999	5	7	ns
≥$60,000	18	51	ns
Ethnicity			
White, non-Hispanic	16	48	ns
White, Hispanic	2	4	ns
Black, non-Hispanic	0	1	ns
Black, Hispanic	1	0	ns
Asian	3	5	ns
Native American	1	1	ns

*ns* not significant.

^a^Fisher’s exact test.

^b^*t*-test with Welch’s correction for unequal variances.

### Sex-specific metabolomic features in human milk for infants with risk of future NDD

**Fig. 2:**
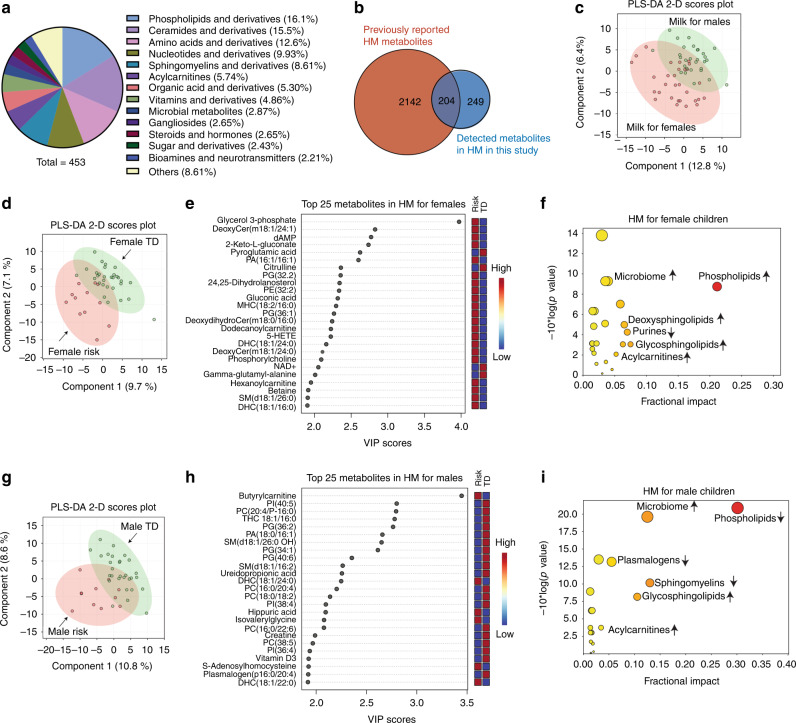
Early biosignatures in human milk (HM) metabolome for children with the risk of future neurodevelopmental delay (NDD) compared with typically developing (TD) children. **a** Metabolites and their chemical classes detected in the HM samples in this study. **b** The comparison of HM metabolome coverage between previously reported milk metabolites and the metabolites detected in this study. **c** The HM metabolomic profiles differed substantially according to offspring sex (*n* = 29 for male TD and 30 for female TD infants). **d** Partial least square discriminant analysis (PLS-DA) based on the HM metabolome data separates female infants with the future NDD risk (*n* = 12) from female TD children (*n* = 30). **e** The top 25 differential metabolites in the HM between the female risk and female TD groups in the PLS-DA model ranked by variable importance in projection (VIP) scores. **f** The metabolic pathways altered in the HM metabolome for female children in the risk group compared to those in the TD group. Increase (upper arrows) and decrease (down arrows) in the risk group. **g** PLS-DA reveals dramatically different metabolic phenotypes in the HM for male children between the risk (*n* = 11) and TD (*n* = 29) groups. **h** The top 25 differential metabolites in the HM for male infants between the risk and TD groups in the PLS-DA model ranked by VIP scores. **i** Pathway impact analysis of altered biochemical pathways in HM for male children with higher risk of future NDD. Upper arrows: increase in the risk group. Down arrows: decrease in the risk group.

We performed the comprehensive characterization of the human milk metabolome using a multi-platform, targeted, broad-spectrum approach. The representative chromatograms for the metabolomic analysis of an HM sample are shown in Supplementary Fig. S1, and the raw data are reported in Supplementary Table S4. A total of 453 endogenous metabolites were detected in the HM samples with phospholipids as the most abundant chemical classes, followed by ceramides, amino acids, nucleotides, sphingomyelins, and acylcarnitines (Fig. 2a). Organic acids, vitamins, microbial metabolites, gangliosides, steroids, sugars, amines, and neurotransmitters were also detected. We compared these detected HM metabolites in our study with the existing HM metabolome data and found that 249 metabolites were reported for the first time in the HM samples in our study (Fig. 2b and Supplementary Table S5). Furthermore, partial least square discriminant analysis (PLS-DA) revealed distinct differences in HM metabolome between male and female TD infants (Fig. 2c). HM for males had higher levels of virtually all the lipid classes we measured than those for the females, including phospholipids, sphingomyelins, cardiolipins, plasmalogens, and acylcarnitines (Supplementary Fig. S2a, b). We therefore analyzed male and female infants separately to identify the metabolic biosignatures in HM for infants with the risk of future NDD compared with TD infants.

In the PLS-DA 2D plot of the metabolomic results (Fig. 2d), the female risk children had strong early chemical signatures in their HM metabolome that were distinct from the TD controls. The HM metabolites that separate female risk from female TD children in the PLS-DA model were ranked by their variable importance in projection (VIP) scores in Supplementary Table S6, and the top 25 most discriminating metabolites are shown in Fig. 2e. Pathway impact analysis showed that lipid abnormalities constituted about 63% of the top 8 pathway changes in females (Supplementary Table S7). Compared with female TD children, the most striking change in lipid pathways was the increase of phospholipids, glycosphingolipids, deoxysphingolipids, and acylcarnitines in HM for female infants at risk for future NDD (Fig. 2f). The top non-lipid changes included the decrease of purines and the increase of microbiome metabolites (Fig. 2f). Similarly, PLS-DA showed distinct metabolomic differences in HM for the male infants between children with and without NDD risk (Fig. 2g). The discriminating HM metabolites between the male TD and male risk group in the PLS-DA model were ranked by their VIP scores in Fig. 2h and Supplementary Table S8. Males were also dominated by lipid abnormalities, which account for about 72% of the top 8 pathway changes (Supplementary Table S9 and Supplementary Fig. S3). Glycosphingolipids and acylcarnitines were increased, while phospholipids, sphingomyelins, and plasmalogens were decreased in the HM for male risk infants (Fig. 2i). In the non-lipid pathways, most microbiome metabolites (6 out of 7) were increased in the male risk group (Fig. 2i and Supplementary Table S8).

Even though 13 biochemical pathways changes in HM metabolome were common to both male and female infants with the risk of future NDD, the direction of change was opposite for 7 pathways between sexes (Supplementary Fig. S4). For example, phospholipids and sphingomyelins were found to be increased in HM for females at risk. In contrast, these metabolites were decreased in HM for males at risk. Nineteen metabolic pathways were altered in only one sex, among which deoxysphingolipids were increased in the HM for females at risk, and plasmalogens were decreased in the HM for males at risk (Supplementary Fig. S4).

### Human milk exposome and exposome-metabolome correlations

**Fig. 3:**
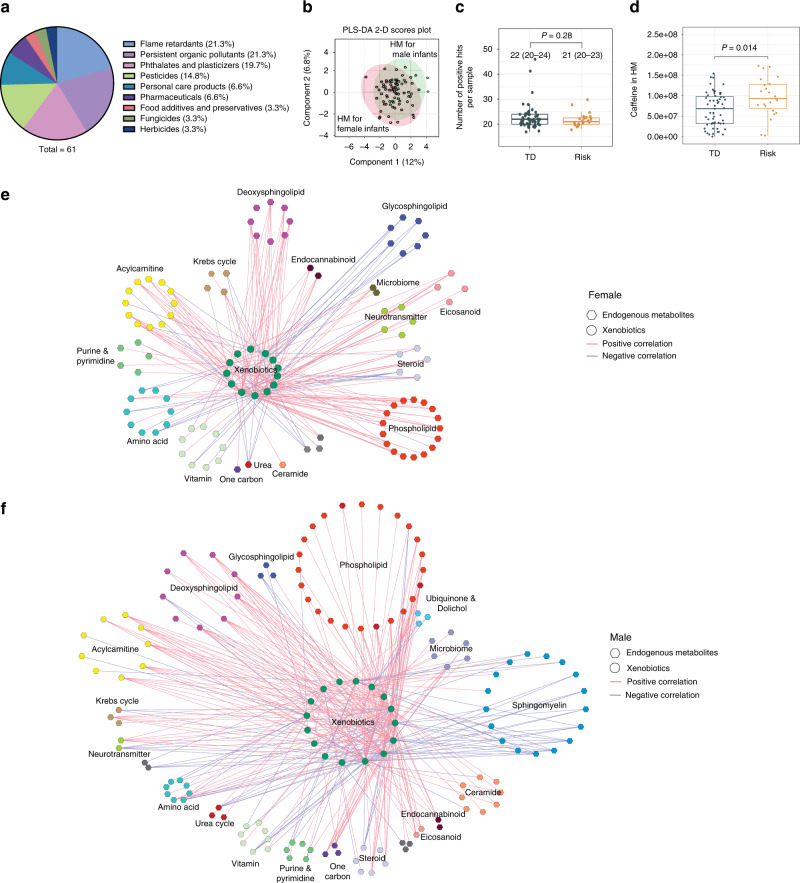
Human milk (HM) exposome and metabolome × exposome correlations. **a** A pie chart shows the chemical class breakdown of the 61 detected xenobiotics out of 937 anthropogenic compounds screened in the HM samples in this study. **b** Partial least square discriminant analysis (PLS-DA) demonstrates the large overlap of HM exposome profiles between male (*n* = 40) and female infants (*n* = 42). **c** The number of positive hits per HM sample was not significantly different between children with the risk of neurodevelopmental delay at the median age of 13.5 months (risk group, *n* = 23) and typically developing children (TD group, *n* = 59). **d** The concentration of caffeine in the HM of the risk group (*n* = 23) was significantly higher than that in the TD group (*n* = 59). Mann–Whitney *U* test was conducted. **e**, **f** The metabolome × exposome correlation networks in milk for female infants (**e**, *n* = 30) and male infants (**f**, *n* = 29). Only correlations with Spearman’s rank correlation *r* values > 0.5 or <−0.5 and *q* values < 0.05 are shown in the networks.

We next characterized the intrinsic human milk exposome to explore the xenobiotics and their metabolomic responses in HM associated with the risk of NDD in the offspring. Out of 937 common anthropogenic compounds screened (Supplementary Tables S2 and S3), 61 were detected in our HM samples and confirmed with the purified standards. These included flame retardants, persistent organic pollutants, phthalates, pesticides, personal care products, pharmaceuticals, food additives, and others (Fig. 3a and Supplementary Fig. S5). There were no significant sex differences in HM exposome between male and female infants (Fig. 3b). The median number of positive hits per sample was 20 (IQR: 20–24) in HM for TD infants and 21 (IQR: 20–23) for infants with future NDD risk (*P* = 0.28) (Fig. 3c). Interestingly, we found a significantly higher level of caffeine in HM for the children with NDD risk than that in the TD group (*P* = 0.014, Fig. 3d).

We then explored the correlations between HM exposome and the metabolome to explore the metabolomic responses to external exposures. As shown in Fig. 3e and Supplementary Table S10, the exposome in the HM for female infants had significant positive correlations with the endogenous metabolites in the biochemical pathways of phospholipid, deoxysphingolipid, eicosanoid, acylcarnitines, and negative correlations with glycosphingolipids (Spearman’s correlation *r* > 0.5 or <–0.5 and *q* value < 0.05). Besides the positive correlations with phospholipid and deoxysphingolipid metabolism as observed in the female group, the HM exposome for male infants was positively correlated with ceramides and negatively correlated with sphingomyelins (Fig. 3f and Supplementary Table S11).

### Multi-analyte models for early prediction of the risk of neurodevelopmental delay

We used the combination of data-driven approaches using ML methods and knowledge-based methods (biochemical pathway analysis) to select the optimal features from HM metabolome and exposome for predicting the future risk of NDD in the offspring (Supplementary Fig. S6). Metabolomic and exposomic data are highly dimensional, and ML can reduce the dimensionality of omics data and improve the risk prediction accuracy.^34^ Random forest (RF) analysis is a common ML method for omics-based predictive models due to its resilience to high dimensionality, insensitivity to noise, and robustness to overfitting.^35^ We used the RF algorithm to identify which variables have a more determinant impact on the prediction outcomes. The importance of the variables in HM metabolome and exposome contributed to the classification between the risk and TD group was ranked by mean decrease accuracy scores in Supplementary Table S12 for female infants and Supplementary Table S13 for males. PLS-DA was used for feature reduction and kNN clustering analysis was performed to help reduce the feature redundancy (Supplementary Tables S14 and S15, Supplementary Figs. S7 and S8). Mann–Whitney *U* test was further applied to verify the results of ML. Finally, pathway enrichment analysis was conducted, and the optimal features were refined to cover different biochemical pathways (Supplementary Figs. S9 and S10).

**Fig. 4:**
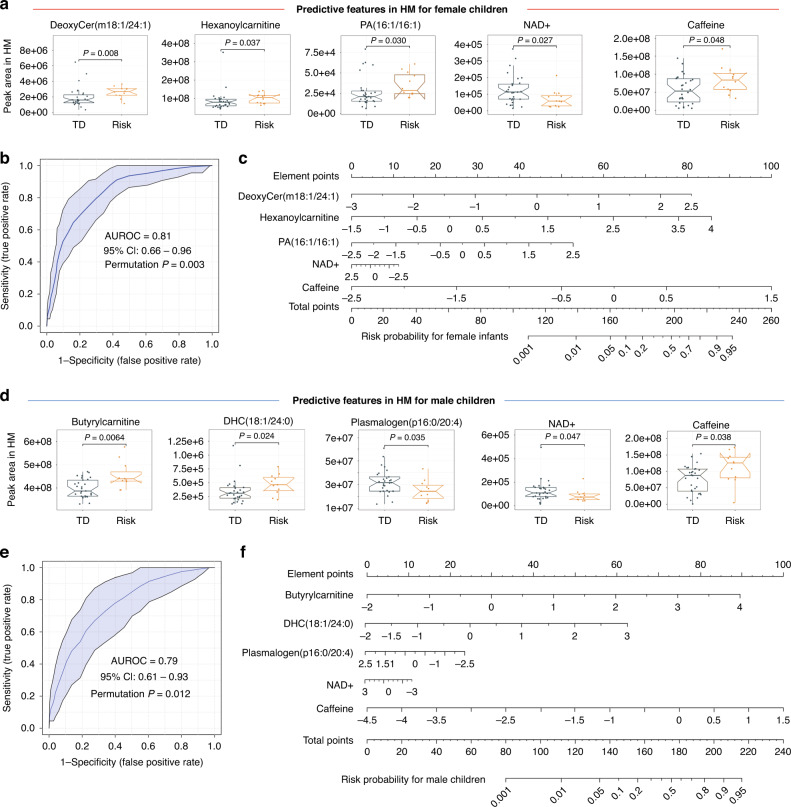
Multi-analyte models based on the human milk (HM) metabolome and exposome predict the children’s future neurodevelopmental delay risk. **a** The selected analytes in the predictive model for female infants. The notched box plots represent the minimum and maximum values (whiskers), the IQR (length of the box), and the 95% CI around the median (notches). *n* = 12 for the risk group and *n* = 30 for the TD group. **b** The ROC curve for female infants. **c** The nomogram for predicting the risk of NDD in females using the selected analytes from HM metabolome and exposome. *Z*-scores were used. **d** The selected predictive analytes for male children. *n* = 29 for the male TD group and 11 for the male risk group. **e** The ROC curve for male children. **f** The nomogram for predicting the risk of future NDD in male infants using the *Z*-scores of the selected analytes.

Figure 4a shows the group differences of the selected analytes in the model for predicting NDD risk for female infants. DeoxyCer (m18:1/24:1), hexanoylcarnitine, PA (16:1/16:1), and caffeine were significantly higher in HM for female infants in the risk group than those in the TD group and negatively correlated with ASQ2 scores (Fig. 4a and Supplementary Fig. S11a). In contrast, the NAD+ level was significantly lower in the HM samples for the risk children compared with TDs and positively correlated with ASQ2 scores. ROC analysis yielded a AUROC of 0.81 (95% CI: 0.66–0.96, permutation *P* = 0.003) (Fig. 4b). In addition, the developed predictive model had the sensitivity of 83.3% (95% CI: 51.5–97.9), specificity of 76.7% (95% CI: 57.7–90.1), accuracy of 78.6% (95% CI: 63.2–89.7), and MCC of 0.55 for predicting future NDD risk for female infants (Table 2). A nomogram was then developed to provide a quantitative method for predicting the individual risk of NDD in females (Fig. 4c). The nomogram maps the predicted risk probabilities into element points on a scale from 0 to 100 in a user-friendly graphical interface. The element point for each metabolite in the model is calculated based on its *Z*-score. For example, an HM sample with DeoxyCer (m18:1/24:1) of −1 (*Z*-score) will be assigned to 30 element points (the upper scale in Fig. 4c). The total points accumulated by all covariates in the nomogram correspond to the predicted probability of NDD risk for a child.

**Table 2:** Performance analysis of the multi-analyte discriminator in the human milk metabolome and exposome for predicting future neurodevelopmental delay.

Multi-analyte predictive model	Sensitivity (%) (95% CI)	Specificity (%) (95% CI)	Accuracy (%) (95% CI)	Matthews correlation coefficient (MCC)
Female infants	83.3 (51.6–97.9)	76.7 (57.7–90.1)	78.6 (63.2–89.7)	0.55
Male infants	72.7 (39.1–93.9)	79.3 (60.3–92.1)	77.5 (61.5–89.2)	0.49

*N* = 30 for female TD infants and 12 female risk infants, and *N* = 29 male TD infants and 11 male risk infants. Values were calculated based on 2 × 2 contingency table analyses. The predictive model for female infants contains five metabolites from the human milk (HM), including DeoxyCer (m18:1/24:1), Hexanoylcarnitine, PA (16:1/16:1), NAD+, and caffeine. The predictive model for male infants contains six analytes: Butyrylcarnitine, SM (d18:1/26:0 OH), DHC (18:1/24:1), plasmalogen (p16:0/20:4), NAD+, and caffeine.

A risk prediction model for males was also developed using the selected optimal features (Fig. 4d). Butyrylcarnitine, DHC (18:1/24:0), and caffeine were significantly higher in HM for female infants in the risk group than those in the TD group and negatively correlated with ASQ2 scores (Fig. 4d and Supplementary Fig. S11b). In contrast, the levels of plasmalogen (p16:0/20:4), and NAD+ were significantly lower in the HM samples for the male risk children compared with TDs and positively correlated with ASQ2 scores. The predictive performance evaluated using ROC analysis showed an AUROC of 0.79 (95% CI: 0.61–0.93, permutation *P* = 0.012) (Fig. 4e). Other performance metrics for the predictive model for males are listed in Table 2 and the nomogram is shown in Fig. 4f.

### Sample size estimation for validation study

We estimated the sample size required for a future large validation study by Pearson’s correlation-based effect size (variance explained) *r* of 0.25. In the current discovery phase study, 22 metabolites in the female group, and 34 metabolites in the male group had absolute *r* values > 0.25, which represents a medium to large effect size. Based on these calculations, a total of 124 females and 124 males, with 62 children at risk and 62 TD controls from each sex, will be required for a future validation study.

## Discussion

To our knowledge, this is the first metabolomics and exposomics study to demonstrate an early signature in human milk for infants at risk of future NDD. The primary patterns of metabolic alterations we found include critical metabolites involved in myelination and brain development, such as deoxysphingolipids, acylcarnitines, sphingomyelins, phospholipids, and glycosphingolipids, including several globosides and cerebrosides. Predicting cognitive outcomes based on infant assessments is known to be challenging because problems such as language impairment, learning disabilities, and social-emotional disorders emerge over time and cannot be evaluated accurately at an early age.^3^ In this study, by the combination of ML methods and knowledge-based approaches, we successfully developed sex-specific, multi-analyte predictive models from the HM metabolome and exposome to predict future risk of NDD in the toddler age with over 75% accuracy. Our models include novel metabolic and exposomic features in HM that are likely to be informative of disease etiology. If replicated, this finding could have a significant impact on clinical practice by permitting infants to be stratified according to their future risk and new preventive treatments to be tested in clinical trials.

Offspring sex-specific differences in human milk composition have been observed for energy content,^36^ total protein,^37^ fat content,^38^ and microbiota diversity.^39^ Beyond the existing literature, in this study, we show dramatic differences in HM metabolome between male and female offspring. HM for male offspring had higher phospholipids, sphingomyelins, cardiolipins, acylcarnitines, and plasmalogens than those in the HM produced for female infants. Moreover, we found that the HM metabolome responds to the infant’s stress differentially between the sexes. Compared to the female TD children, participants with female infants at risk produced more phospholipids in their HM to meet their children’s needs. In contrast, phospholipids in HM for male risk infants were lower than those in the male TD group. Even though the HM exposome did not differ substantially according to the infant sex, the inter-omics analysis revealed significant positive correlations between the xenobiotics and ceramides in HM only for male children.

Low levels of certain critical metabolites in human milk may logically have important effects on infant brain development. In this study, plasmalogens, and NAD+ were observed to be lower in the HM for male infants in the risk group compared to the TD group. Plasmalogens are the main ethanolamine phospholipids in brain myelin and neural membranes, and because of their unique molecular structure, plasmalogens facilitate rapid membrane fusion, which is critical for synaptic neurotransmission.^40^ Our metabolomic analysis confirms the presence of plasmalogens from the phosphatidylethanolamine family in HM, which have been implicated in infant brain development.^41^ Increased HM levels of plasmalogens were reported to be associated with a remarkable increase in brain size in preterm infants.^42,43^ NAD+ is a central redox cofactor of virtually all metabolic processes. Postpartum supplementation of nicotinamide riboside to rodent mothers increased NAD+ content in the rodent milk, leading to improved neurobehavioral development of the offspring, whose advantages persist into adulthood.^44^

Compared to the TD group, we also observed higher levels of some essential metabolites for brain development in mother’s milk for the infants with future NDD risk in a sex-specific manner, including higher phospholipids and sphingomyelins for female risk infants, and higher glycosphingolipids and acylcarnitines for infants in the risk group of both sexes. Interestingly, increased glycosphingolipids, phospholipids, and acylcarnitines are typical metabolic features of cell danger response (CDR).^45^ During the lactation period, the mother and her breastfed offspring maintain a reciprocal regulation of responsiveness to stress in both rodents and human beings.^46^ Activation of CDR represents the mother’s metabolic responses to the offspring’s stress, resulting in the tailoring of breast milk composition to maximize health protection and development for infants at risk.

Caffeine is a methylxanthine that acts as a central nervous system stimulant and naturally occurs in several foods. Neurostimulation occurs because caffeine is an inhibitor of the widely expressed purinergic adenosine receptors 1 and 3 (P1A1 and P1A3, also abbreviated as ADORA1 and ADORA3 receptors). Here, we show that HM for the infants in the risk group had higher caffeine than that for the TD infants. The effects of maternal caffeine consumption on breastfed children are inconclusive, and both negative and positive outcomes have been reported in the literature.^47^ The potential effects of early caffeine exposure on the long-term neurodevelopment of breastfed infants require further investigation.

A limitation of the current study was the relatively small size of the cohort of children at risk. Sample size calculations estimated that at least 124 males and 124 females, with 62 TD and 62 AR of each sex, will be needed for a larger validation phase study. It should also be noted that the nutrient content of HM varies in samples collected at different times of the day.^16,17^ The samples in this study were well matched with a mean sampling time around 1 pm. Another limitation was that the outcome was measured by maternal report using the ASQ at about one year of age, and thus, the assessment of risk for NDD was not diagnostic. However, the ASQ screening instrument is widely used in pediatric practice and can be an important early indicator of future risk, leading to potential further evaluation, ongoing monitoring, or specialized intervention.

## Conclusion

In summary, multi-omics profiling of the maternal milk metabolome and exposome revealed significant differences between children at risk for neurodevelopment delay (NDD) and typically developing (TD) infants. Other well-known risk factors such as prematurity, low birth weight, perinatal difficulties, and socioeconomic variables were well controlled and were unable to account for the differences between groups in this study. The early biosignature in human milk for infants who later screened positive for NDD was dominated by the sex-specific abnormalities in deoxysphingolipids, sphingomyelins, plasmalogens, phospholipids, and acylcarnitines. Multi-analyte models of the biosignature of milk samples collected before 9 months of age showed over 75% accuracy in predicting NDD documented nearly one year later, raising the possibility that milk metabolomics might provide an early warning of later developmental risk. Caution is warranted in interpreting these results. This was a small pilot study. Larger validation studies will be needed to determine the sensitivity, specificity, cost/benefit, and clinical practicality of milk metabolomics as a tool in pediatrics. However, if validated in larger studies, milk metabolomics might one day be used as another early screening tool to help identify infants at risk of NDD with the aim of instituting treatment before the first symptoms appear.

## Supplementary information

Supplementary materials

Supplementary Table S1

## Data Availability

The raw metabolome and exposome data for this study are provided in Supplementary Tables S3 and S4. In-house python tools used in this study can be found on GitHub (https://github.com/BDNav/PeakVetting).
