# Supplementary material for: Metabolomic and exposomic biomarkers of risk of future neurodevelopmental delay in human milk
Source: Pediatr Res. 2022 Sep 15;93(6):1710–20. doi: 10.1038/s41390-022-02283-6 (PMC10172108; doi:10.1038/s41390-022-02283-6)
Supplement: Supplementary file 1 — Supplementary materials [file 41390_2022_2283_MOESM1_ESM.docx]

**SUPPLEMENTARY MATERIALS**

**Metabolomic and Exposomic Biomarkers of Risk of Future Neurodevelopmental Delay in Human Milk**

Kefeng Li^1,2^, Kerri Bertrand^3^, Jane C. Naviaux^1,5^, Jonathan M. Monk^6^, Alan Wells^3^, Lin Wang^1,2^, Sai Sachin Lingampelly^1,2^, Robert K. Naviaux^1-4*^, Christina Chambers^3,7*^

^1^The Mitochondrial and Metabolic Disease Center, Departments of ^2^Medicine, ^3^Pediatrics, ^4^Pathology, ^5^Neurosciences, ^6^Bioengineering, ^7^Herbert Wertheim School of Public Health and Human Longevity Science, University of California, San Diego School of Medicine, San Diego, CA, USA.

*Corresponding authors:

Robert K. Naviaux: E-mail:[rnaviaux@health.ucsd.edu](mailto:rnaviaux@health.ucsd.edu);

Christina Chambers: E-mail: [chchambers@health.ucsd.edu](mailto:chchambers@health.ucsd.edu)

**SUPPLEMENTARY METHODS**

**Preparation of commercial isotopically labeled internal standard mix**

L-Glutamine (^15^N2, 98%) (Cat# NLM-1328), Glycine (1-^13^C, 99%; 15N, 98%) (Cat# CNLM-507), L-Aspartic acid (^13^C_4_, 99%) (Cat# CLM-1801), labeled acyl-carnitine standards (Cat# NSK-B and NSK-B-G1), Adenine (8-^13^C, 95%) (Cat# CLM-1654), D-Sucrose (^13^C_12_, 98%) (Cat# CLM-7757), PC (16:0/16:0)-d_62_ (Cat# DLM-11099) and Ceramide (d18:1/16:0)-d31 (Cat# CLM-9582) were purchased from Cambridge Isotope Laboratories (MA, USA). Creatinine-(methyl-^13^C) (Cat# 488615) was obtained from Sigma Aldrich. The stable isotope standards were diluted using 100% methanol and mixed for further use.

**Preparation and calibration of custom-synthesized** **uniformly ^13^C labeled stable isotopes**

Uniformly labeled stable isotope internal standards were custom-synthesized in the yeast (*Komagataella phaffii*, ATCC, Cat# 76273) by metabolic labeling with ^13^C_6_-glucose (Cat# 389374, Sigma Aldrich, 99.5%) and ^13^C-sodium formate (Cat# 279412, Sigma Aldrich, 99%). Briefly, the yeast strain was cultured at 30 ˚C in M9 minimal media supplemented with ^13^C_6_-glucose and ^13^C-sodium formate as the sole carbon source to achieve an OD_600_ of 3 - 4. The cells were harvested by centrifugation, and uniformly ^13^C labeled metabolites were extracted from the pellets by adding 4 volumes of cold (-20 ˚C) methanol-acetonitrile (50:50, v/v). The suspension was vortexed, incubated on ice for 10 min, vortexed again, then cellular debris and macromolecules were removed by centrifugation at 16,000 g for 10 min at 4 ˚C. The supernatants containing ^13^C labeled metabolites were then frozen at -80 ˚C until use. Each batch of custom-synthesized standards was calibrated by determining the percent ^13^C enrichment of leucine, uridine, adenosine, AMP, glycerol-3-phosphate, palmitoyl-carnitine, and selected phosphatidylcholine lipids and sphingomyelins.

**Extraction of endogenous metabolites and xenobiotics from HM**

The HM samples were thawed on ice and carefully mixed by means of inversion. Ninety (90) µL of HM samples was spiked with 5 µL of commercial internal standards and 5 µL of custom-synthesized ^13^C labeled standards. The preparation of commercial and custom-synthesized isotopically labeled reference standards is described in the Supplemental Methods. Four volumes (400 µL) of 100% ethanol prechilled at -20 °C was added and vortexed, then incubated on ice for 10 min, and centrifuged for 10 min at 16,000 g at 4 °C. The supernatants containing the extracted metabolites, xenobiotic compounds, and internal standards were transferred to labeled cryotubes and stored at -80 °C for further analysis.

**Metabolome profiling**

Targeted, broad-spectrum, metabolomic analysis of endogenous metabolites was performed using a UFLC XR HPLC system (LC-20AD, Shimadzu) coupled with a Qtrap 5500 triple quadrupole mass spectrometer (SCIEX) (LC-MS/MS). We performed LC-MS/MS analysis in both hydrophilic interaction liquid chromatography (HILIC-MS/MS) mode and reverse phase (RP-MS/MS) mode. A total of 659 endogenous metabolites covering all major human metabolic pathways and diverse chemical classes were targeted, including 553 metabolites in HILIC-MS/MS mode and 106 metabolites in RP-MS/MS.

**HILIC-MS/MS.** Ten (10) µL of the ethanol extract was injected through a CTC PAL autosampler and separated on a polymer-based NH2 column (250 × 2 mm, 4 µm) (Asahipak NH2P-40 2E, Shodex). The LC conditions were as follows: Mobile phase A: 95% H_2_O with 20 mM (NH_4_)_2_CO_3_ and 5% ACN, pH 9.8. Mobile phase B: 100% ACN. The gradient was: 0 - 3.5 min 95% B, 3.6 - 8 min 85% B, 8.1 - 13 min 75% B, 14 - 30 min 0% B, 31 - 41 min 95% B, 41.1 min stop. The flow rate was 200 µL/min, and the column temperature was held at 25 ºC. The MS/MS detection was performed using electrospray ionization (ESI) and by advanced scheduled multiple reaction monitoring (MRM) with dynamic windowing. The ESI source conditions were set as follows: electrospray voltage of -4500V for negative mode and 5500V for positive mode, source temperature of 500 °C, curtain gas of 30, ion source gas 1, and gas 2 of 35 psi, respectively. Compound-dependent parameters were optimized using the purified standards.

**RP-MS/MS.** Ten (10) µL of the ethanol extract was injected through a CTC PAL autosampler and separated on a Raptor Biphenyl column (150 × 2.1 mm, 2.7 µm) (Restek). The LC conditions were as follows: Mobile phase A: 90% H_2_O with 0.1% formic acid and 10% MeOH, pH 4.0. Mobile phase B: MEOH-IPA (50:50, v/v) with 0.1% formic acid. The gradient was: 0 - 2 min 10% B, 2.1 - 4 min 40% B, 4 -12 min, linear ramping up to 100% B, 12 – 18 min 100% B, 19 - 24 min 10% B, 24.1 min stop. The flow rate was 250 µL/min, and the column temperature was controlled at 40 ºC. The MS/MS detection was performed using ESI and by advanced scheduled MRM. The ESI source conditions were set as follows: electrospray voltage of -4500V for negative mode and 5500V for positive mode, source temperature of 500 °C, curtain gas of 30, ion source gas 1, and gas 2 of 35 psi, respectively.

**Quality control (QC)**

Two levels of reproducibility were used daily before passing metabolomic results for data analysis-sample QC and platform QC. In the first level, stable isotope-labeled internal standards were added to every biological sample, extracted, and the peak shape, retention time shift, and peak area variability of spiked stable isotope labeled internal standards were inspected in each sample. Samples that failed this QC analysis were reinjected the next day. In the second level of quality control, HPLC and mass spec instrument stability were assessed using 4 replicate injections per day of a standardized lot of pooled human plasma containing stable isotope labeled internal standards (SIL-ISDs). The AUCs of 59 representative metabolites (13 SIL-ISDs and 46 endogenous compounds) in the replicate QC samples were monitored daily for platform process control. Reproducibility was quantified by calculating the within-day and within-day plus between-day Pearson correlations and median of the relative standard deviations (RSDs). The intra-day and inter-day Pearson correlations for QC injections were typically ≥ 0.999 and 0.998, respectively. The intra-day and inter-day median RSDs for 59 representative metabolites in replicated QC injections on 3 days were 7.0% (IQR 4%-14%) and 6.0% (IQR 3%-13%), respectively.

**Exposome profiling**

Exposome profiling of xenobiotic chemicals in HM was conducted using both LC-MS/MS in reverse phase mode (RP-MS/MS) and gas chromatography (GC)-MS/MS-based on the chemical properties of the targets.

RP-MS/MS analysis was conducted using a Shimadzu LC-20AD UHPLC system coupled with a SCIEX Qtrap 5500 MS/MS. Ten µL of ethanol extract was injected and separated using a Raptor Biphenyl column (150 × 2.1 mm, 2.7 µm) (Restek). The LC conditions were as follows: Mobile phase A: 90% H_2_O with 0.1% formic acid and 10% MeOH, pH 4.0. Mobile phase B: MEOH-IPA (50:50, v/v) with 0.1% formic acid. The gradient was: 0 - 2 min 10% B, 2.1 - 4 min 40% B, 4 -12 min, linear ramping up to 100% B, 12 – 18 min 100% B, 19 - 24 min 10% B, 24.1 min stop. The flow rate was 250 µL/min, and the column temperature was controlled at 40 ºC. The MS/MS detection was performed using electrospray ionization (ESI) and by advanced scheduled multiple reaction monitoring (MRM). The ESI source conditions were set as follows: electrospray voltage of -4500V for negative mode and 5500V for positive mode, source temperature of 500 °C, curtain gas of 30, ion source gas 1, and gas 2 of 35 psi, respectively.

GC-MS/MS analysis was performed using an Agilent 8890 GC coupled with a 7010B triple quadrupole mass spectrometer. One µL of ethanol extract was injected in splitless mode with an ultra-inert inlet liner (Agilent, Catalog: 5190-2203), and the inlet temperature was kept at 280 ºC. The GC separation was performed using two fused silica HP-5MS UI capillary columns of 15 m × 0.25 mm (inner diameter), 0.25 µm (thickness) (Agilent) connected by a pneumatic switching device (PSD) to facilitate backflushing between injections. High purity helium (99.999%) was used as the carrier and quench gas, and nitrogen was used as the collision gas. The oven temperature was programmed as follows: 60 ºC for 1 min; 40 ºC /min to 120 ºC, and then 5 ºC /min to 310 ºC. Retention time locking was performed using chlorpyrifos-methyl as a standard, and flow rates were adjusted to achieve a retention time lock of 18.1 min. The triple quadrupole was operated in electron ionization (EI) mode, and the temperature for the transfer line was set at 310 ºC. The EI ion source was operated at 280 ºC for source temperature and 150 ºC for quadrupole temperature. The dynamic multiple reaction monitoring (dMRM) was used for data acquisition with a gain factor of 10 and the solvent delay of 3 min. Backflushing was conducted after the analytical run at 310 ºC and 6 mL/min flow. The total run time was 40 min, followed by 5 min of backflushing, and 5 minutes for full oven and column cooldown.

A total 937 common anthropogenic compounds were targeted, including 145 analytes on the RP-MS/MS platform and 792 chemicals on the GC-MS/MS platform. MRM transitions and the retention time (RT) were optimized using the purified standards. Two MRMs transitions were used for each analyte if available. The complete targeted list and the associated MS/MS parameters and RT are listed in the Supplementary Table S1 and Table S2.

**Statistical analysis**

**Metabolomics data analysis and biochemical pathway enrichment**

Metabolomic data were log_2_ transformed and scaled by control SDs prior to statistical analysis. There were no missing values, and no data were imputed for the metabolome dataset. Partial least square analysis (PLS-DA) was conducted in MetaboAnalyst 5.0. Metabolites with variable importance in projection (VIP) scores ≥ 1.5 in the PLS-DA model were considered significant. Metabolic pathway analysis was performed using a custom python script with an in-house library for human metabolism. Significant metabolites were grouped into biochemical pathways by the sum of their VIP scores to determine the rank-ordered significance of each pathway (fractional impact). The hypergeometric test was used to evaluate whether a particular metabolite set was represented more than expected by chance within the given library.

**Exposomics data analysis**

Peaks were initially screened using in-house python peak vetting scripts according to their peak area, RT, signal to noise (S/N) ratios, and sample to blank ratios. The filtering criteria for peaks from LC-MS/MS were sample to blank ratios ≥ 10 for both MRM transitions. The cut-off criteria for peaks from GC-MS/MS were as follows: (1) S/N ratio ≥ 3 for MRM1; (2) S/N ratio ≥ 2 for MRM2; (3) RT difference ≤ 0.2 min; (4) Sample to blank ratio ≥ 10 for MRM1; (5) Sample to blank ratio ≥ 3 for MRM2; (6) Peak area for MRM1 ≥ 2000. Putative positive hits were manually inspected and verified by the purified standards. Compounds with > 25% missing values were removed for statistical analysis. The rest of the missing values were imputed by probabilistic principal component analysis (PPCA) with the same probabilistic distribution of the observed data. After PPCA, The data were then log2 transformed and scaled by control SDs, and the resulting Z-scores were used for further analysis.

**Dimensional reduction and feature selection by machine learning**

Three machine learning algorithms, including PLS-DA, random forest (RF), and k-nearest neighbor (KNN), were applied to reduce the input variables for the predictive models using R 4.0.5 with corresponding R packages. RF was conducted with 5000 trees. Biochemical pathway analysis was also undertaken to guide the section of features from different metabolic pathways.

**SUPPLEMENTARY DISCUSSION**

**Strengths of this study**

Our study has several strengths. First, we performed a comprehensive metabolomic and exposomic analysis of HM to systematically search for a potential early signature of future risk for neurodevelopmental delay. Second, we analyzed the omics data and generated robust predictive models by the combination of several data-driven, advanced machine learning algorithms and knowledge-based pathway analysis. The selected sex-specific predictors reflect both the basic chemical composition of HM and the maternal responses to the offspring’s stress. Third, the known confounding variates were well controlled and balanced between the risk and the TD group, such as infant age, maternal age, maternal BMI, birth weight, and gestational age at delivery.

**SUPPLEMENTARY TABLES (as a single Excel file)**

**Supplementary Table S1.** Participant and sample characteristics.

**Supplementary Table S2.** List of xenobiotics measured by LC-MS/MS.

**Supplementary Table S3.** List of xenobiotics measured by GC-MS/MS.

**Supplementary Table S4.** The raw metabolome and exposome data for human milk in this study.

**Supplementary Table S5.** Metabolites previously reported in cow or human milk and measured in our study.

**Supplementary Table S6.** Metabolites and xenobiotics in human milk that separate female infants with the future risk of neurodevelopmental delay (the risk group) from typically developing female infants (the TD group) in the PLS-DA model.

Notes: Date are ranked by the variable importance in projection (VIP) scores. n = 30 for female TD and n = 12 for the female risk group.

**Supplementary Table S7.** The significantly altered metabolic pathways in human milk metabolome for female infants with the future risk of neurodevelopmental delay compared with the female TD infants.

**Supplementary Table S8.** Metabolites and xenobiotics in human milk that separate male infants with the risk of future neurodevelopmental delay (male risk group) from typically developing male infants (male TD group) in the PLS-DA model.

Notes: Data are ranked by the variable importance in projection (VIP) scores. n = 29 for male TD and n = 11 for the male risk group.

**Supplementary Table S9.** The significantly altered metabolic pathways in human milk metabolome for male infants with the risk of future neurodevelopmental delay compared with the male TD infants.

**Supplementary Table S10.** The significant metabolome × exposome correlations in human milk for female typically developing (TD) infants (n = 30).

Notes: Missing values in exposome data were replaced using the probabilistic principal component analysis (PPCA). Spearman's correlation analysis was performed using the log2 transformed data. Correlation coefficient r values > 0.5 or < -0.5, and q values < 0.05 were considered as statistically significant.

**Supplementary Table S11.** The significant metabolome × exposome correlations in human milk for male typically developing (TD) infants (n = 29).

Notes: Missing values in exposome data were replaced using the probabilistic principal component analysis (PPCA). Spearman's correlation analysis was performed using the log2 transformed data. Correlation coefficient r values > 0.5 or < -0.5, and q values < 0.05 were considered as statistically significant.

**Supplementary Table S12.** Metabolites and xenobiotics in human milk contribute to the separation of female infants with the future risk of neurodevelopmental delay (female risk) from typically developing female infants (female TD) in the random forest model.

Notes: Data are ranked by mean decrease accuracy (MDA) scores (n = 5000 trees). n = 30 for female TD and n = 12 for the female risk group’.

**Supplementary Table S13.** Metabolites and xenobiotics in human milk contribute to the separation of male infants with the risk of neurodevelopmental delay (male risk) from typically developing male infants (male TD) in the random forest model.

Notes: Data are ranked by mean decrease accuracy (MDA) scores (n = 5000 trees). n = 29 for male TD and n = 11 for the male risk group.

**Supplementary Table S14.** The kNN and k-means clusters of human milk metabolome and exposome for female infants.

**Supplementary Table S15.** The kNN and k-means clusters of human milk metabolome and exposome for male infants.

**SUPPLEMENTARY FIGURES**


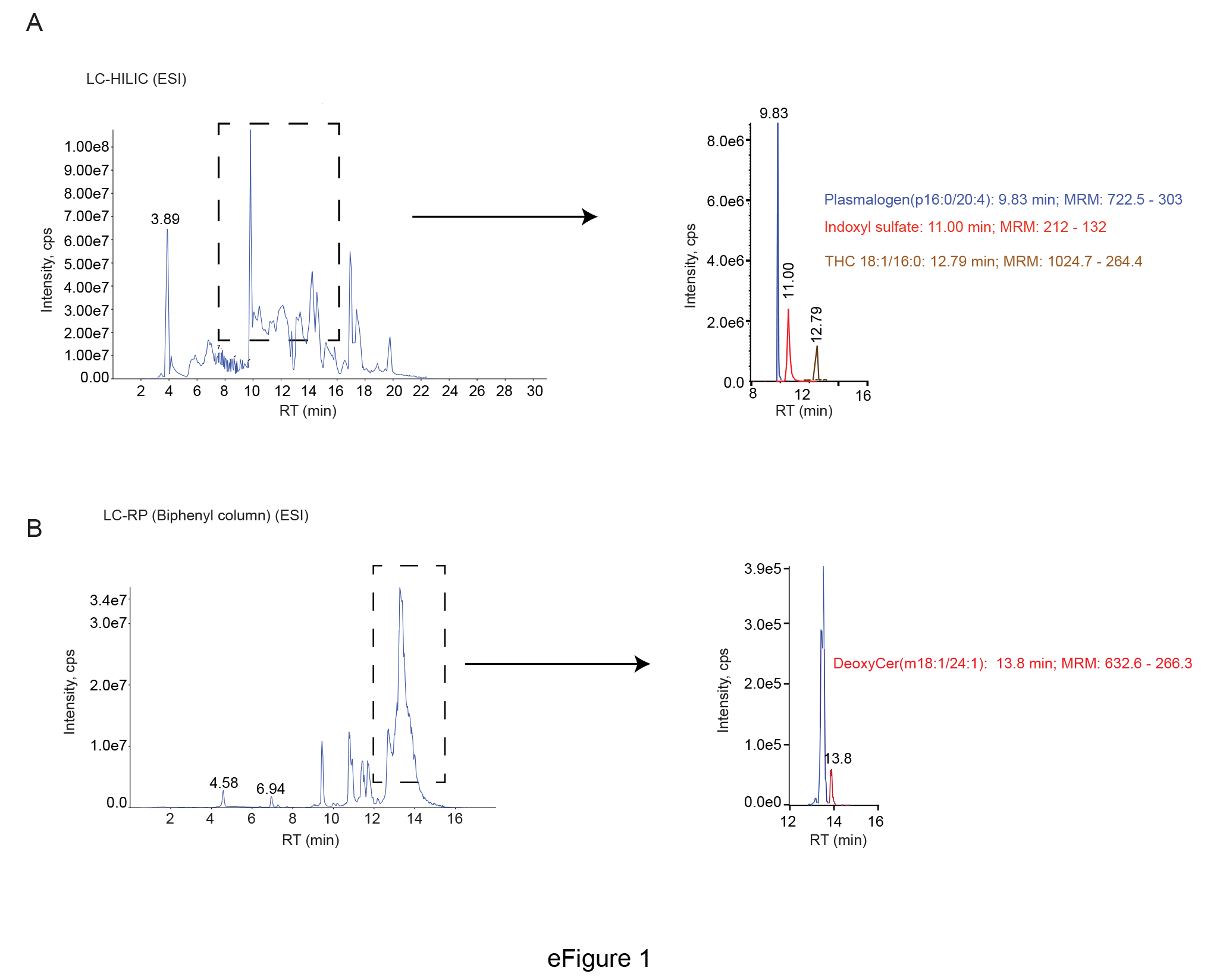


**Supplementary Fig. S1.** The metabolomic total ion chromatograms (TICs) and representative metabolites of a human milk sample. (A) A hydrophilic interaction liquid chromatography (HILIC) run. (B) A reverse-phase run on the Biphenyl raptor column.


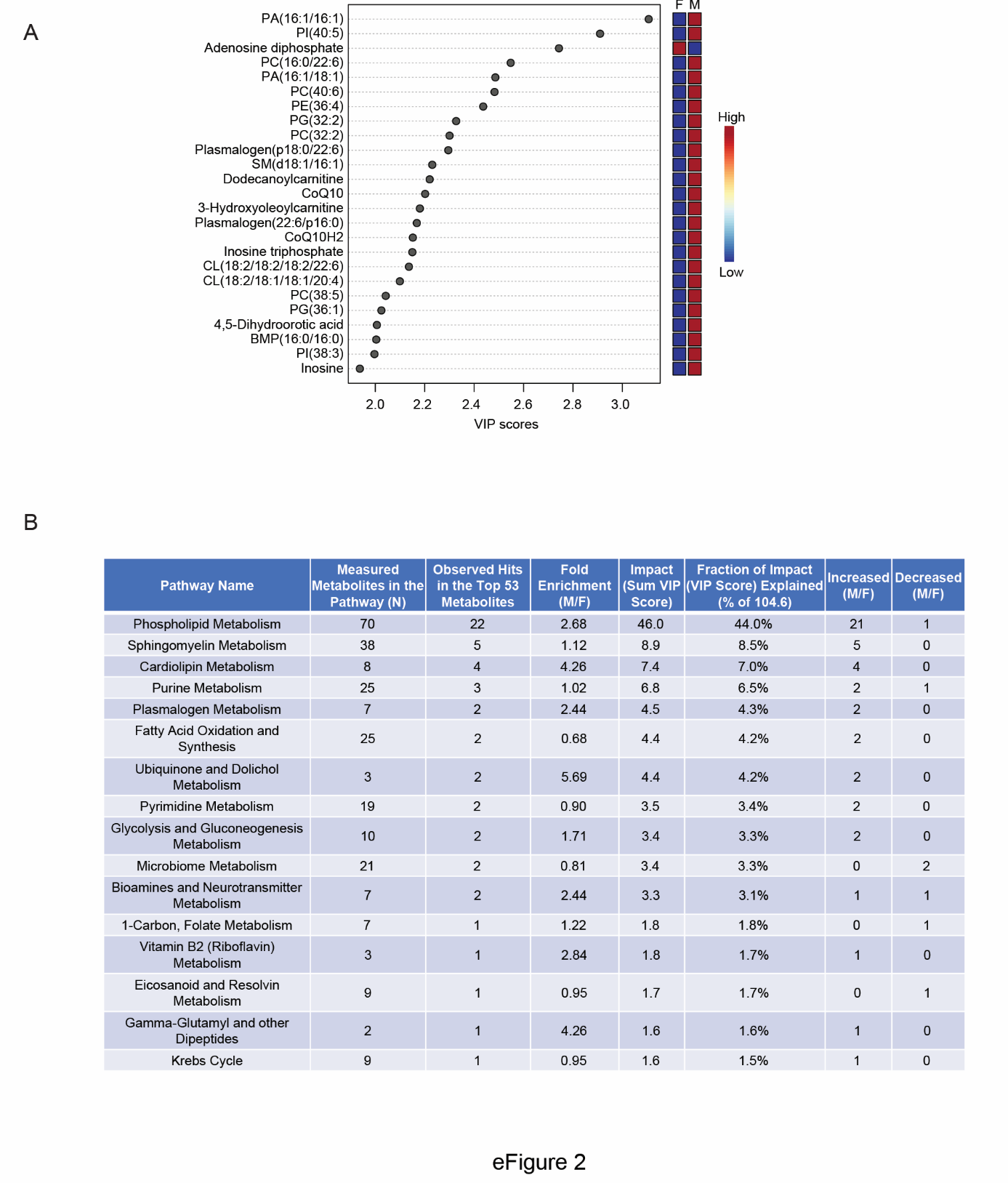


**Supplementary Fig. S2.** The differential metabolites and their biochemical pathways in human milk for male and female offspring. **(**A) Top 25 differential metabolites ranked by variable importance in projection (VIP) scores. VIP ≥ 1.5 was considered as statistically significant. (B) The differential metabolic pathways in human milk for male and female children. n = 29 for male TD infants and 30 for female TD infants.


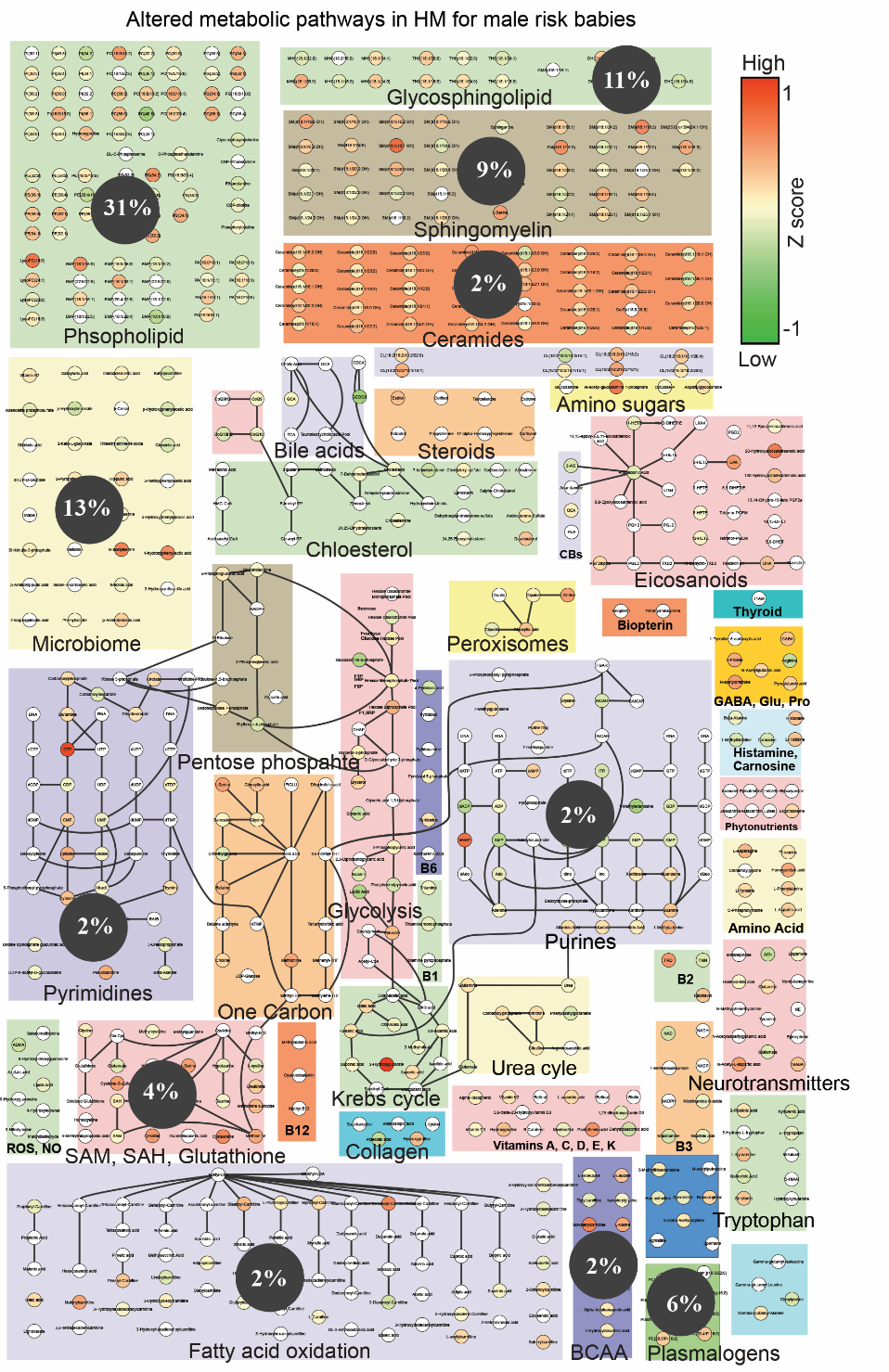


**Supplementary Fig. S3.** Cytoscape visualization of the changes of metabolic pathways in human milk for children with the risk of future neurodevelopmental delay.

Notes: The fractional contribution of each pathway is indicated as a percentage of the total variable importance in projection (VIP) score in black circles. The smaller circles indicate the measured metabolites in each pathway, quantified by z-scores. Metabolites in red were increased, and those in green were decreased in the human milk for children with future neurodevelopmental risk compared to the typically developing children.


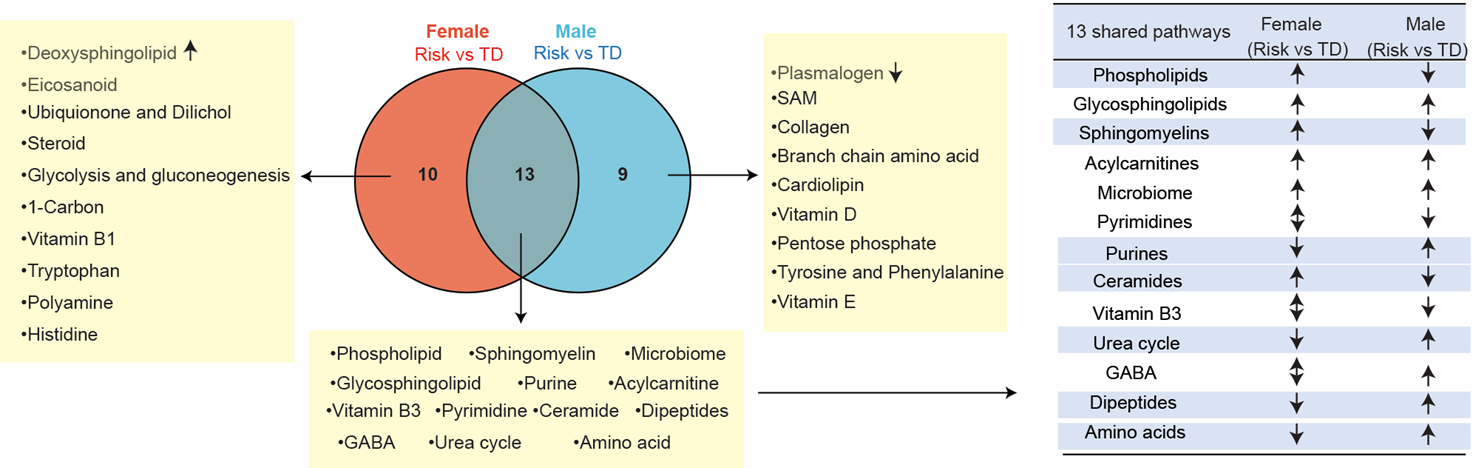


**Supplementary Fig. S4.** Shared and unique metabolic pathways altered in HM metabolome for male and female infants in the risk group.

Upper arrows indicate the increase in the risk group and down arrows indicate the decrease in the risk group. The bidirectional arrows indicate the mix of increased and decreased metabolites in the pathway.


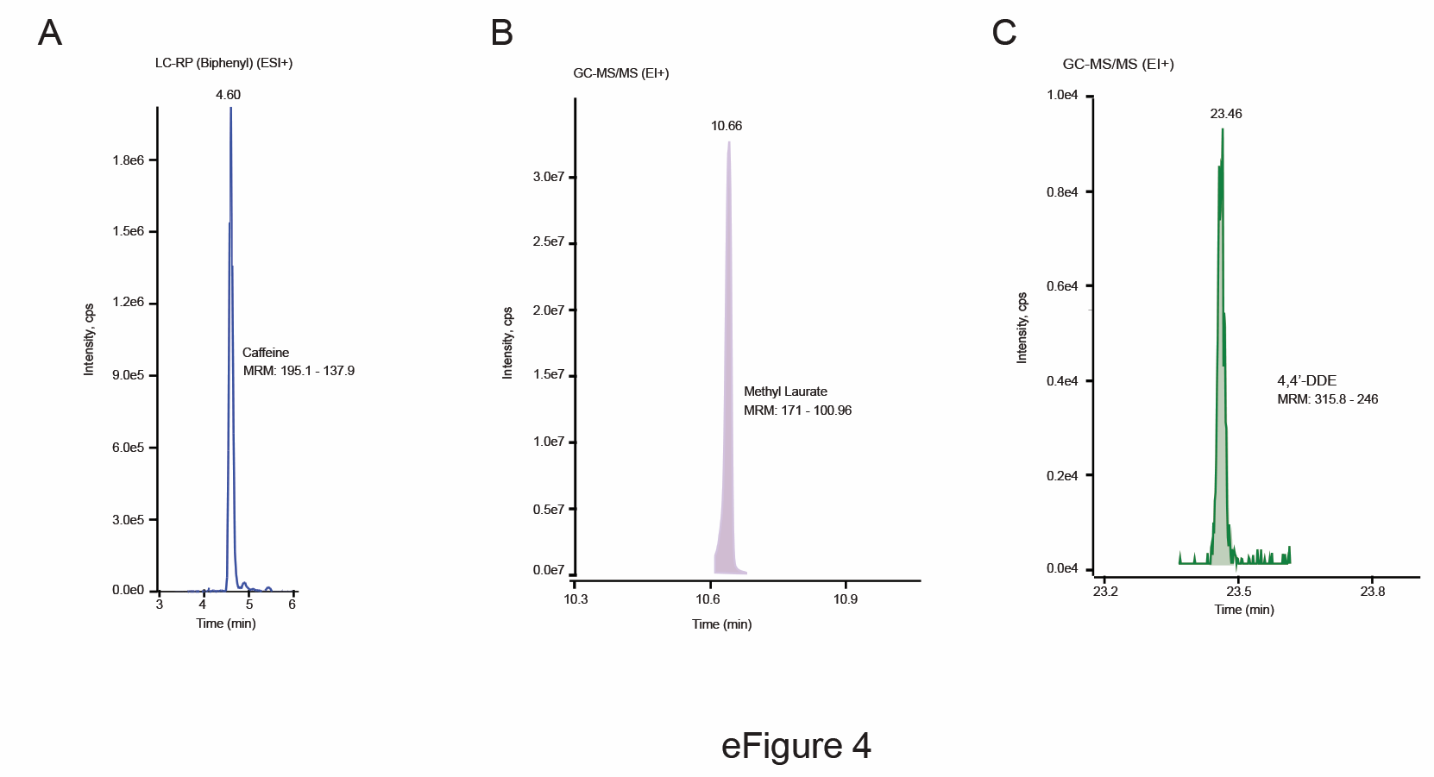


**Supplementary Fig. S5.** The representative xenobiotics detected in the human milk samples.


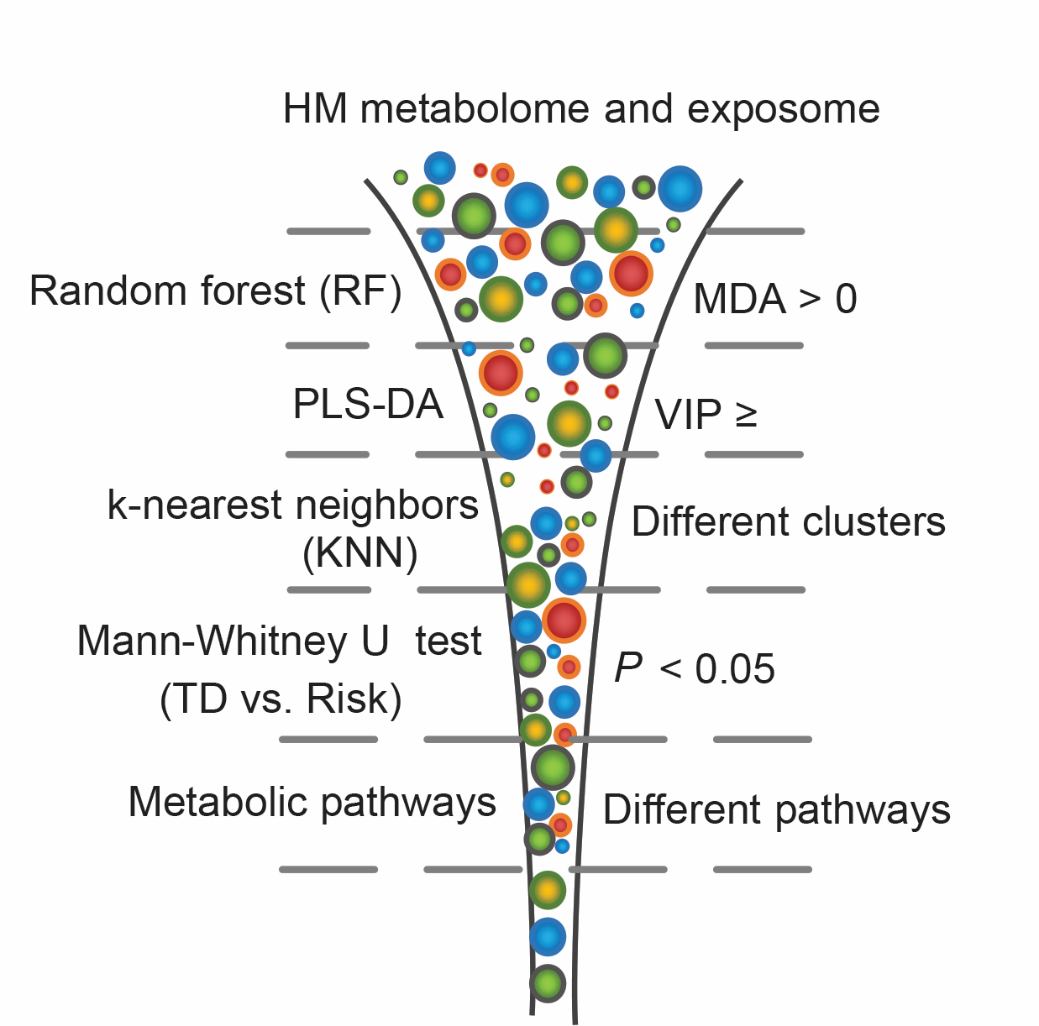


**Supplementary Fig. S6.** The flow chart of feature selection using the combination of data-driven and knowledge-driven methodologies.

Abbreviation: MDA: Mean decrease accuracy, PLS-DA: Partial least square analysis, VIP: variable importance in projection.


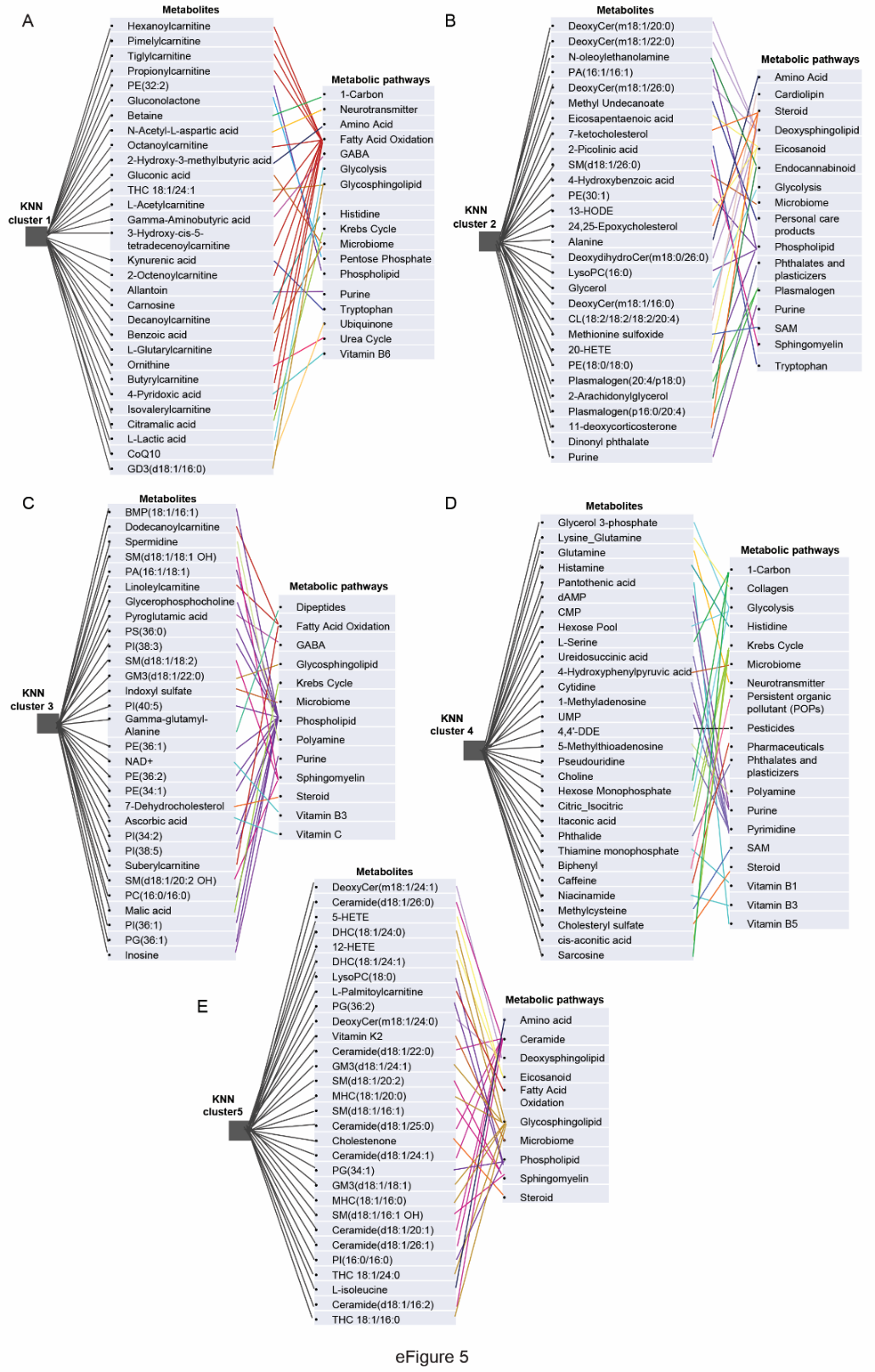


**Supplementary Fig. S7.** The clusters of metabolites and their metabolic pathways identified by the k-nearest neighbor (kNN) algorithm in human milk for female infants. The top 30 metabolites ranked by their mean decrease accuracy (MDA) scores (random forest, 5000 trees) were shown in each cluster.


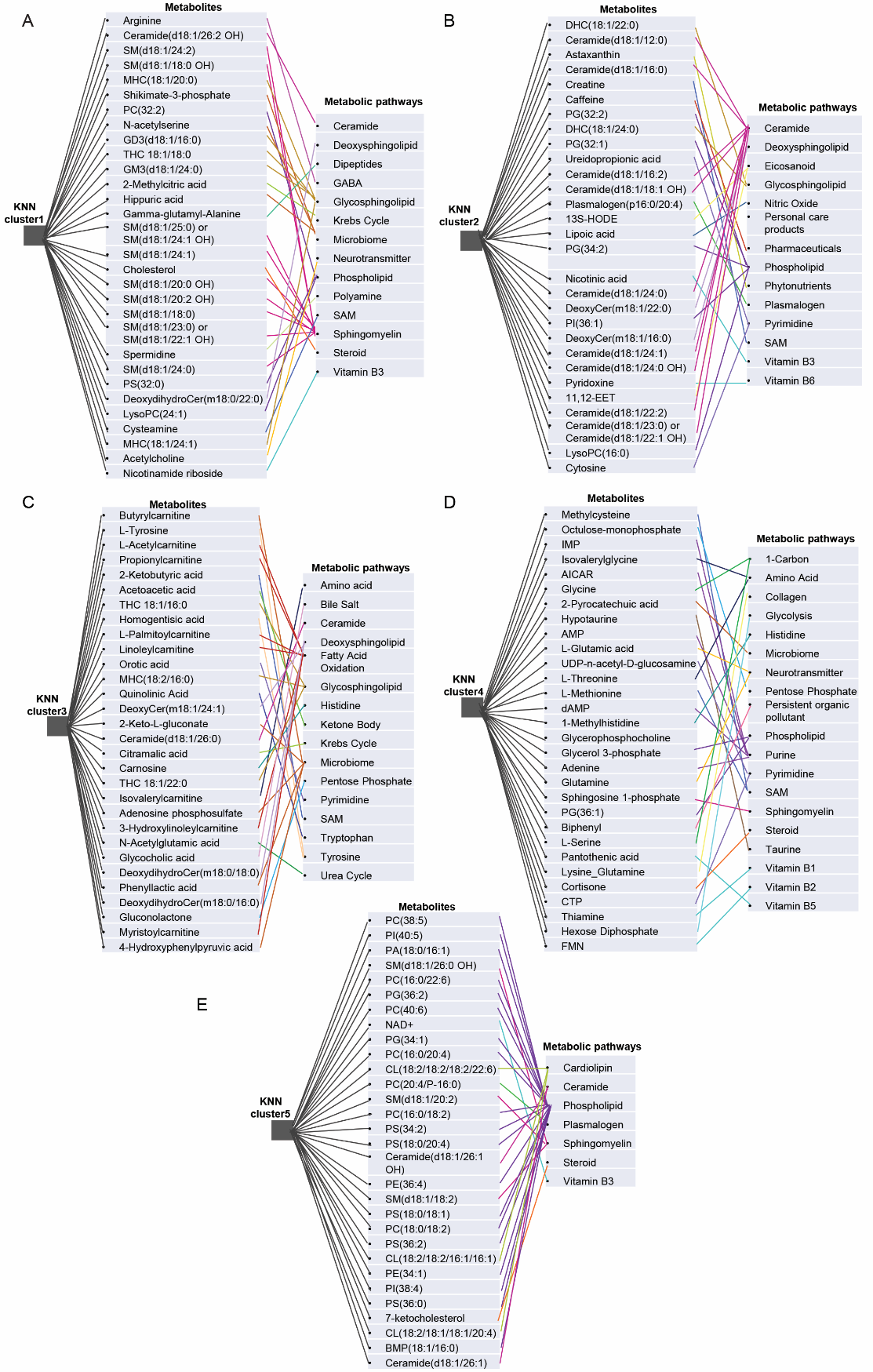


**Supplementary Fig. S8.** The clusters of metabolites and their metabolic pathways identified by the k-nearest neighbor (kNN) algorithm in human milk for male infants. The top 30 metabolites ranked by their mean decrease accuracy (MDA) scores (random forest, 5000 trees) were shown in each cluster.


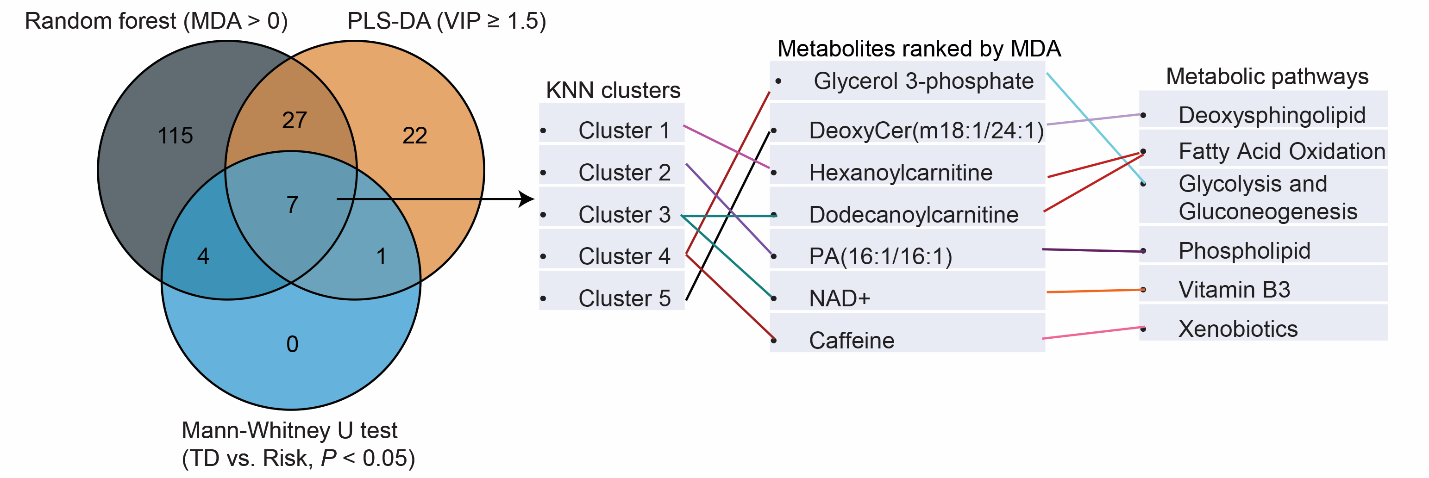


**Supplementary Fig. S9.** The selection of optimal features from HM metabolome and exposome for predicting the risk of future neurodevelopmental delay in female children.


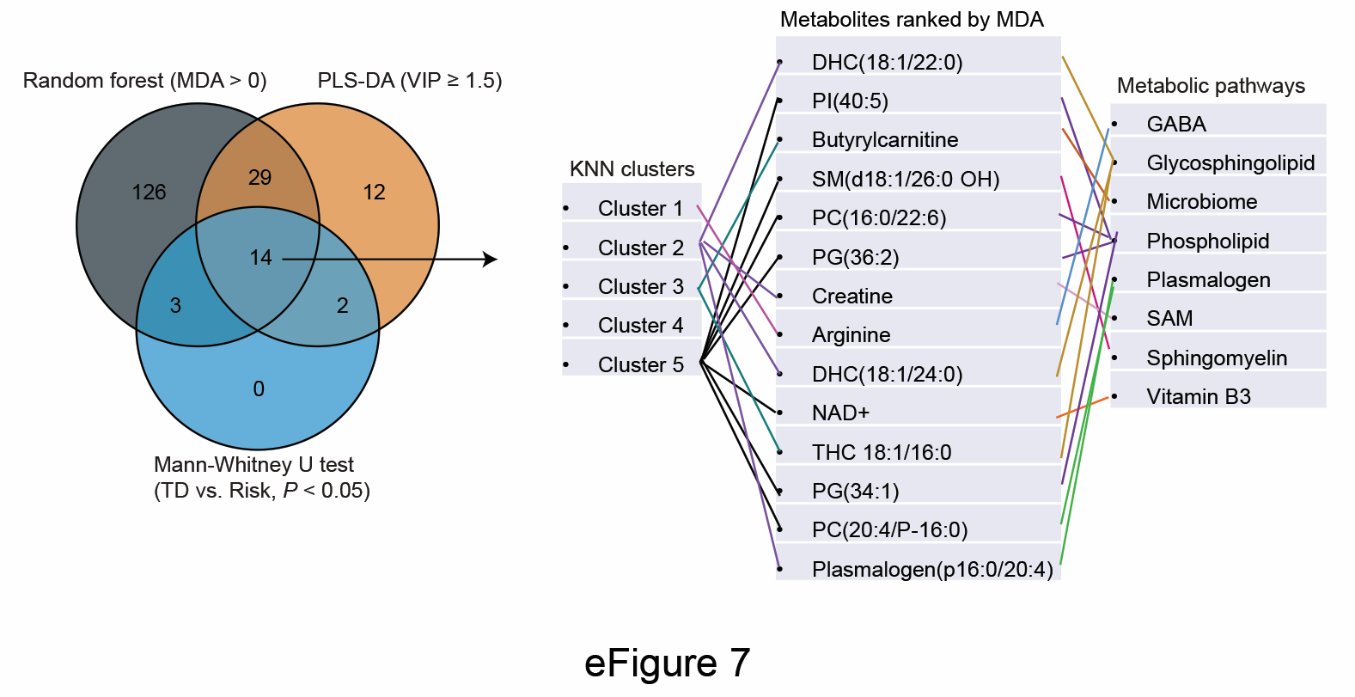


**Supplementary Fig. S10.** The selection of optimal predictive features from the metabolome and exposome of maternal breast milk for predicting the future neurodevelopmental delay risk in males.


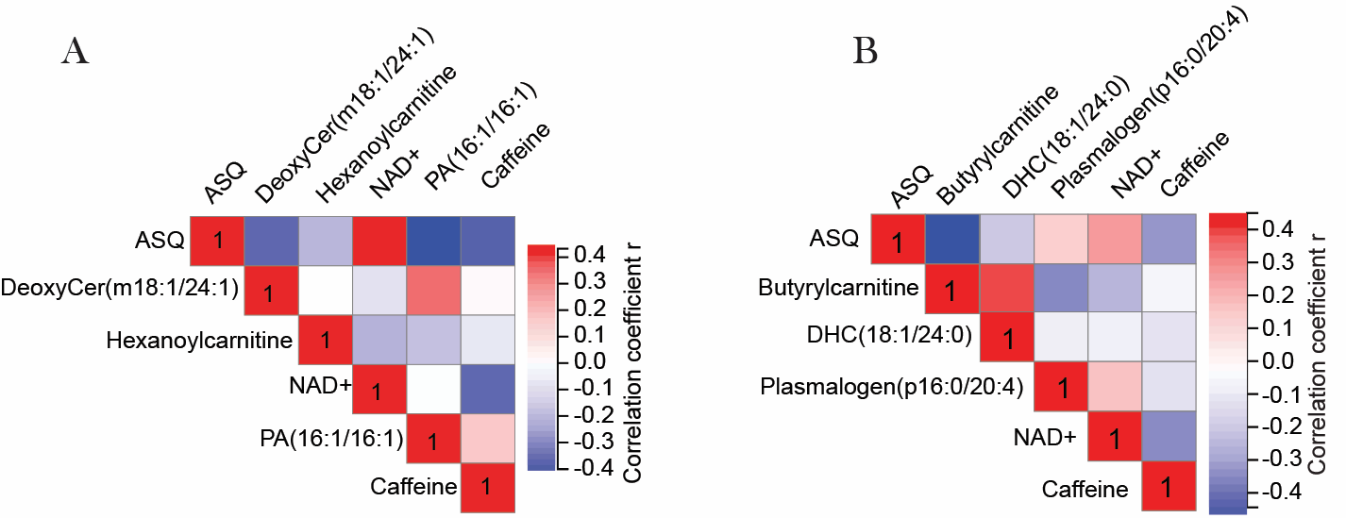


**Supplementary Fig. S11.** The correlations between the normalized Ages and Stages Questionnaire-2 (ASQ-2) scores and the abundance of the selected metabolite predictors in the HM. The peak areas were log2 transformed, and Spearman’s rank correlation was then performed. (A): Female; (B): Male.
